# 

**Published:** 1917-09-01

**Authors:** 


### CONTENTS.

| | PAGE |
|---|---|
| **The Army Medical Service Inquiry** ... ... | 429 |
| **The Disinfection of Sick-Rooms** ... ... ... | 430 |
| **Hospital and Institutional News** ... ... .. | 431 |
| Orthopædics in the West Riding—American War Hospitals—Wounded in France and their Friends—A Ward on Wheels—Well-known Leicester Surgeons Honoured—Patients' Prejudices—Workmen's Representation at Merthyr—Bridgend Military Hospital—Macclesfield Infirmary and Anonymous Critics—The Pensions Appeal Tribunal—The Business Side of Cripples' Workshops—County Visitation Society—Addenbrooke's Hospital, Cambridge—First-hand Information for Friendly Societies—The Determination of Sex—The Welsh National Memorial Association—This Week's Drug Market—To Our Readers. | |
| **The Worst War of the World** ... ... ... | 435 |
| IV. The Germans' Barbarities and the World's Press. | |
| **Physical Methods in Deformity and Disease** | 437 |
| Some Hindrances to their More Extended Employment. | |
| **Parliament and the Profession** ... ... ... | 440 |
| **The Institutional Library** ... ... ... ... | 441 |
| Sex Determination in Man. | |
| **The Two New Orders** ... ... ... ... | 443 |
| Many Honours for Hospital Workers. | |
| **Nursing Affairs in Ireland** ... ... ... ... ... | 444 |
| The Main Irish Grievance—Economic. | |
| **The Matrons' and Sisters' Department** ... | 445 |
| Matrons in Council: V. The Elements Must be in the Woman. | |
| Round the Hospitals. | |
| Nurse Sweating under Charity's Cloak—The Withheld Certificate—Is Four Years' Delay Sweating?—Australian Government's Referee Answers Yes—Adelaide Hospital's Patriotic Example—How the College Helps Nurses. | |
| **Food in Wartime** ... ... ... ... ... ... | 448 |
| Rational Economy. | |
| **A Jungle Hospital** ... ... ... ... ... ... | 449 |
| II. Causes of Plague, Former Residencies, and Some Sport. | |
| **The Editor's Letter-Box** ... ... ... ... | 450 |
| The Society of Women Journalists. Physical Orthopædics. | |
| **Legal Notes for Institutional Workers** ... | iv |
| Working of the Venereal Disease Act. The Medical Man as County Court Assessor. | |
| **Matrons' and Sisters' Appointments** ... ... | iv |
| **Personalia** ... ... ... ... ... ... ... | iv |
| **The Institutional Worker** ... ... (Suppl.) | 1 |
| Smoking in Hospital: Answer to August Question. September Question. Questions Invited. Enquiries and Answers. | |

